# “Screening for small-for-gestational age neonates at early third trimester in a high-risk population for preeclampsia”

**DOI:** 10.1186/s12884-020-03167-5

**Published:** 2020-09-25

**Authors:** Raquel Mula, Eva Meler, Sandra García, Gerard Albaigés, Bernat Serra, Elena Scazzocchio, Pilar Prats

**Affiliations:** 1 Department of Obstetrics, Gynecology and Reproduction, Hospital Universitari Dexeus, Dexeus Mujer, Barcelona, Spain; 2 Hospital Clinic de Barcelona, Institut Clínic de Ginecologia Obstetrícia i Neonatologia, Barcelona, Spain; 3 Institut Català de la Salut, Atenció a la Salut Sexual i Reproductiva (ASSIR) de Barcelona, Barcelona, Spain

**Keywords:** SGA neonates, high-risk patients, uterine artery Doppler, placental growth factor, prediction

## Abstract

**Background:**

Strategies to improve prenatal detection of small-for-gestational age (SGA) neonates are necessary because its association with poorer perinatal outcome. This study evaluated, in pregnancies with first trimester high risk of early preeclampsia, the performance of a third trimester screening for SGA combining biophysical and biochemical markers.

**Methods:**

This is a prospective longitudinal study on 378 singleton pregnancies identified at high risk of early preeclampsia according to a first trimester multiparametric algorithm with the cutoff corresponding to 15% false positive rate. This cohort included 50 cases that delivered SGA neonates with birthweight < 10th centile (13.2%) and 328 cases with normal birthweight (86.8%). At 27–30 weeks’ gestation, maternal weight, blood pressure, estimated fetal weight, mean uterine artery pulsatility index and maternal biochemical markers (placental growth factor and soluble FMS-Like Tyrosine Kinase-1) were assessed. Different predictive models were created to evaluate their performance to predict SGA neonates.

**Results:**

For a 15% FPR, a model that combines maternal characteristics, estimated fetal weight, mean uterine artery pulsatility index and placental growth factor achieved a detection rate (DR) of 56% with a negative predictive value of 92.2%. The area under receiver operating characteristic curve (AUC) was 0.79 (95% confidence interval (CI), 0.72–0.86). The DR of a model including maternal characteristics, estimated fetal weight and mean uterine artery pulsatility index was 54% (AUC, 0.77 (95% CI, 0.70–0.84)). The DR of a model that includes maternal characteristics and placental growth factor achieved a similar performance (DR 56%, AUC 0.75, 95% CI (0.67–0.83)).

**Conclusions:**

The performance of screening for SGA neonates at early third trimester combining biophysical and biochemical markers in a high-risk population is poor. However, a high negative predictive value could help in reducing maternal anxiety, avoid iatrogenic interventions and propose a specific plan for higher risk patients.

## Background

Growth-restricted fetuses are at increased risk of perinatal mortality and morbidity, but the risks can be reduced when the condition is prenatally detected. The traditional approach of identifying pregnancies at high risk of delivering low-weight neonates is measurement of symphysis–fundus height, with a low detection rate of affected fetuses, not higher than 30% [1]. A routine third-trimester ultrasound examination with measurement of fetal biometries is superior in identifying pregnancies at high risk of delivering low-weight neonates, with detection rates around 50% [2, 3] but the impact in improving perinatal outcome is unclear [4]. The last meta-analysis concluded that routine ultrasound in low-risk or unselected population did not confer any benefit on the mother nor the baby in terms of perinatal mortality, preterm birth less than 37 weeks, caesarean section rates, and induction of labour rates [5].

The terms small-for-gestational age (SGA) and fetal growth restriction (FGR) have often been used interchangeably, but not all small neonates are growth-restricted, and not all growth-restricted neonates are small. Therefore, identification of high-risk neonates only by estimating fetal weight by ultrasound may be missing a significant proportion of cases with latent placental insufficiency. It seems reasonable to find new strategies to improve the detection of these neonates, especially when it is well known that SGA neonates have been associated with poorer perinatal outcome [6].

Maternal epidemiological characteristics such as maternal history of chronic hypertension and an impairment of placental function indirectly assessed by uterine Doppler measurement play a crucial role in the pathogenesis of placental-related pathology [7, 8]. Moreover, some studies have demonstrated changes in maternal plasma concentration of angiogenic and anti-angiogenic factors in these patients who will end up with preeclampsia (PE) and/or an SGA neonate [9]. Combined screening approaches at first trimester have been shown to be useful for the prediction of early-onset PE [10] but there is less evidence of its utility for late events, such us late PE, SGA neonates or adverse perinatal outcome, that are more prevalent. Since the screening of these entities is one of the major goals of current maternal-fetal medicine, new approaches to the prediction of late onset pregnancy complications have been proposed. Some studies have evaluated third trimester strategies incorporating maternal parameters and placental biomarkers and their improvement in the detection rate of SGA fetuses in the absence of preeclampsia [11–13]. Since most of these studies are based in general population and the two conditions share pathophysiological mechanisms, is interesting to evaluate the risk of SGA neonates in a subgroup of patients identified at high risk of PE early in the pregnancy.

The aim of our study was to evaluate, in a subgroup of women classified as high risk of early preeclampsia using a first trimester multiparametric algorithm, the performance of a contingent third trimester screening for SGA combining maternal characteristics, uterine Doppler, fetal biometry, mean arterial pressure and biochemical markers.

## Methods

### Study design, setting and data source

Between 2014 and 2016, a prospective longitudinal study was undertaken at the Fetal Medicine Unit in the Hospital Universitari Dexeus, Barcelona (a tertiary university teaching hospital). The protocol was approved by the Dexeus Institutional Review Board for Human Investigation and the Ethics Committee (23/3/2013, reference number 20130313/17). Written informed consent was obtained from all patients and the reported investigations were carried out in accordance with the principles of the Declaration of Helsinki as revised in 2008. Data about pregnancy follow-up and outcome were collected from the electronic patient history.

### Study population

All pregnancies were singletons and had complete follow-up in our center. Recorded patient characteristics included maternal age, method of conception (spontaneous or by assisted reproduction technique (ART)), cigarette smoking during pregnancy, maternal pathology such as history of chronic hypertension, pre-existing diabetes mellitus (DM), thrombophilia, kidney and autoimmune diseases, parity and obstetric history recording previous pregnancy with PE or SGA neonate.

### Follow-up

Patients were enrolled at 8–9 weeks of pregnancy. Maternal history, height (cm) and weight (kg) were recorded and an ultrasound exploration was done concomitantly to date the pregnancy and confirm an ongoing singleton pregnancy at this moment. Crown-rump length (CRL) was measured in a neutral fetal position and the formula of Robinson and Fleming [14] was used to calculate gestational age. A blood sample was also obtained during this same visit for the determination of placental growth factor (PlGF) and biochemical parameters used for Down screening: free β-human chorionic gonadotropin (free β-hCG) and pregnancy-associated plasma protein A (PAPP-A).

Subsequently, first trimester assessment of the uterine artery Doppler at 11 + 0 to 13 + 6 weeks was performed transvaginally, and the mean uterine artery pulsatility index (UtA PI) was calculated as the average PI between right and left arteries [15]. Maternal weight and blood pressure (BP) were recorded at the time of the first trimester scan. Blood pressure was automatically measured in one arm (right or left), in a sitting position after 5-minute rest, according to the ISSHP recommendations [16]. Mean arterial pressure (MAP) was calculated as: diastolic BP + (systolic BP − diastolic BP)/3. Patients were classified as low or high risk of early PE using the algorithm previously published by Scazzocchio et al. [17]. The former algorithm included maternal characteristics, maternal weight and height, medical history (chronic hypertension, pre-existing diabetes mellitus, thrombophilia, kidney disease and autoimmune disease), obstetric history (previous PE or SGA neonate), MAP and mean UtA PI. High-risk was defined as a cut-off above 1/270, achieving a detection of 96% of cases for a false-positive rate (FPR) of 15% [17].

The high-risk subgroup was followed-up in a specialized high-risk unit. At 19–22, 24–25 and 27–30 weeks of gestation, maternal weight and blood pressure was recorded and a transabdominal scan to measure fetal biometries, estimated fetal weight (EFW), mean UtA PI and umbilical artery (UA) pulsatility index was performed. Fetal biometries (biparietal diameter, head circumference, abdominal circumference and femur length) were measured according to the international guidelines [18]. EFW was calculated using Hadlock’s formula [19] and the centile was derived from local reference curves. If the EFW was below the 10th centile, fetal Doppler was performed: middle cerebral artery (MCA) PI, UA PI and cerebroplacental ratio (CPR). CPR was calculated as the ratio of MCA PI to UA PI. Ductus venosus (DV) PI was performed when previous Doppler parameters (UA, MCA, CPR) were altered. Maternal blood samples for PlGF and soluble FMS-Like Tyrosine Kinase-1 (sFlt-1) were collected at the time of the ultrasound (19–22, 24–25 and 27–30 weeks). SGA was defined as EFW between the 3rd and 10th centiles and normal Doppler and FGR as EFW below 3rd centile or EFW between the 3rd and 10th centile an either abnormal UA PI (> 95th centile), MCA PI (< 5th centile), CPR (< 5th centile) or UtA PI (> 95th centile) [20–22]. Pregnancies with the diagnosis of FGR and SGA fetuses were followed-up according to a specific clinical protocol [23].

### Main outcome

SGA neonates were defined as birth weight less than the 10th centile adjusted by gender and gestational age according to neonatal local standards [24].

### Statistical analysis

Mean ± standard deviation was reported for continuous variables and number and percentage were reported for categorical variables. Some variables transformed into z-score (absolute value minus divided by standard deviation) or MoMs (value divided by median of variable) according to our reference curves.

Chi-square test was used for the association between categorical variables with our primary outcome (SGA). Mann Whitney test or Student’s t-test was performed according to normality hypothesis. In parallel, a logistic regression was fitted to estimate the odds ratio (OR) and the 95% confidence interval (CI).

Finally, to adjust for confounding factors, a multivariable logistic model was analyzed. Receiver operating characteristics (ROC) curve and the area under the curve (AUC) according to sensibility and specificity were used to find the best model to estimate SGA.

All tests were bilateral with a significant level set to 5%. The statistical analysis was performed using IBM SPSS Statistics v22.0 software.

## Results

### Characteristics of the study population and perinatal outcome

The study population included 378 pregnancies with 1st trimester high risk screening of early preeclampsia out of 5876 women with singleton pregnancies screened at first trimester. The incidence of SGA among this cohort was 13.2% (*n* = 50). Our population was 94% Caucasian. Maternal biophysical characteristics, obstetric history and perinatal outcome are presented in Table 1. The incidence of global preeclampsia and gestational hypertension was 6% and 2.1%, respectively.

**Table 1:** Patients’ demographic characteristics and perinatal outcome in pregnancies with normal birthweight or small-for-gestational age neonates

Characteristics	Normal birthweight (*n* = 328)	SGA (*n* = 50)	*P*
Maternal age (years)	35.5 ± 4.5	34.7 ± 4.1	0.239
Maternal weight (kg)	67.8 ± 13.1	61.6 ± 11.4	< 0.001*
Maternal height (cm)	164.9 ± 6.1	163.3 ± 6.7	0.089
BMI (kg/m^2^)	25.0 ± 4.7	23.1 ± 3.7	0.004*
Smoking (%)	7.3 (24)	8 (4)	0.776
Nulliparity (%)	63.1 (207)	78 (39)	0.040*
ART conception (%)	9.8 (32)	4 (2)	0.286
Pre-existing DM (%)	0.3 (1)	0	0.696
Chronic hypertension (%)	4.9 (16)	4 (2)	0.786
Kidney disease (%)	1.2 (4)	0	0.432
Autoimmune disease (%)	4 (13)	2 (1)	0.704
Thrombophilia (%)	1.5 (5)	6 (3)	0.075
Previous PE (%)	4.6 (15)	0	0.236
Previous SGA (%)	2.1 (7)	6 (3)	0.133
Treatment with AAS (%)	11.3 (37)	14 (7)	0.576
GA at delivery	38.8 ± 1.8	38.5 ± 1.8	0.259
Induction of labour (%)	24 (79)	40 (20)	0.017*
GA at induction	39.42 ± 1.62	39.1 ± 1.33	0.226
Cesarean delivery (%)	32 (105)	36 (18)	0.5
Birth weight (mean, g)	3200 ± 493	2498 ± 352	< 0.001*
Neonatal feminine sex (%)	41.2 (135)	32 (16)	0.218
Arterial pH	7.25 ± 0.08	7.27 ± 0.06	0.181
Apgar 5’	9.98 ± 0.64	9.92 ± 0.27	0.995
5’ Apgar score < 7 (%)	0.6 (2)	0	0.580
Arterial pH < 7.15 (%)	10.5 (25)	0	0.055
Preeclampsia (%)	5.8 (19)	8 (4)	0.525
Gestational Hypertension (%)	2.4 (8)	0	0.604

Quantitative variables were expressed as mean ± SD

Categorical variables were expressed as % (n)

(*p < 0.05)

*SGA *Small-for-gestational age, *BMI* Body mass index, *ART* Assisted reproduction technique, *DM* Diabetes mellitus, *PE* Preeclampsia, *GA* Gestational age

When we compared both groups, women with SGA neonates were more frequently nulliparous (78% vs 63.1%, p 0.04) and had lower body mass index (BMI) (23.1 vs 25.0 kg/m^2^, *p* 0.004). The prevalence of smoking (8% vs 7.3%, p 0.776) and treatment with aspirin (14% vs 11%, p 0.576) during pregnancy was similar in both groups. The rate of induction of labor was higher in the SGA group (40% vs 24%, p 0.017) although the gestational age at induction was not statistically different (39.1 ± 1.33 vs 39.42 ± 1.62, p 0.226). The occurrence or preeclampsia (8% vs 5.8%, p 0.525), the rate of cesarean section (36% vs 32%, *p* 0.5), Apgar at 5’ (9.92 ± 0.27 vs 9.98 ± 0.64, *p* 0.995) and arterial pH (7.27 ± 0.06 vs 7.25 ± 0.08, p 0.181) were comparable between the two groups (Table 1).

### First trimester screening

Interestingly, women with SGA neonates had significantly higher mean uterine artery Doppler pulsatility indexes (1.2 ± 0.3 vs. 1.4 ± 0.3, *p* < 0.001) and lower arterial pressure (85.8 ± 7.9 vs. 82.0 ± 7.1, *p* 0.002) at the time of first trimester screening. Moreover, the values PAPP-A (1 ± 0.5 vs. 0.8 ± 0.4, *p* 0.039) and PlGF (0.3 ± 0.2 vs. 0.2 ± 0.2, p0.002) at 8–10 weeks’ gestation were significantly lower (Table 2).

**Table 2:** First (11–13 weeks), second (19–22 weeks) and third (27–30 weeks) trimester evaluation

	Normal birthweight (*n* = 328)	SGA (*n *= 50)	*P*
First trimester evaluation			
- Mean uterine artery PI (MoM)	1.2 ± 0.3	1.4 ± 0.3	< 0.001*
- MAP (mm Hg)	85.8 ± 7.9	82.0 ± 7.1	0.002*
- PAPP-A (MoM)	1 ± 0.5	0.8 ± 0.4	0.039*
- PlGF (MoM)	0.3 ± 0.2	0.2 ± 0.2	0.002*
Second trimester evaluation			
- Mean uterine artery PI (MoM)	1.2 ± 0.6	1.6 ±0.5	< 0.001*
Third trimester evaluation			
- EFW (g)	1399.3 ± 173.2	1244.5 ± 149.0	< 0.001*
- EFW (z-scores)	0.1 ± 1.0	-0.7 ± 0.9	< 0.001*
- Mean uterine artery PI (MoM)	1.2 ± 0.4	1.4 ± 0.5	< 0.001*
- MAP (mmHg)	81.9 ± 10.3	80.5 ± 9.9	0.372
- PlGF (pg/mL)	530.9 ± 423.1	328.4 ± 287.8	< 0.001*
- PlGF (MoM)	1.13 ± 0.9	0.70 ± 0.62	< 0.001*
- sFlt-1 (pg/mL)	1385.5 ± 1118.7	1692.5 ± 1206.7	0.037*
- sFlt-1 (MoM)	0.9 ± 0.7	1.1 ± 0.8	0.037*
- sFlt-1/PlGF	6.2 ± 17.1	14.7 ± 21.7	< 0.001*
- sFlt-1/PlGF (MoM)	1.8 ± 5.2	4.5 ± 6.8	< 0.001*

Quantitative variables were expressed as mean ± SD

(*p < 0.05)

*SGA* Small-for-gestational age neonates, *PI* Pulsatility index, *MoM* Multiples of the median, *MAP* Mean arterial pressure, *PAPP-A* Pregnancy-associated plasma protein A, *PlGF* Placental growth factor, *EFW* Estimated fetal weight, *sFlt-1* Soluble FMS-Like Tyrosine Kinase-1

### Second trimester uterine artery Doppler

Mean uterine artery pulsatility index at 19–22 weeks’ gestation was significantly increased in the group of pregnancies with SGA neonates (Table 2). Interestingly, we found that 48% (25/52) of women with pathological uterine Doppler at first trimester (mean UtA PI > 95th) maintained pathological uterine Doppler at 19–22 weeks’ gestation. Moreover, when we evaluated the group of women with SGA neonates, that percentage increased to 73.3% (11/15).

### Third trimester screening

The mean gestational age at evaluation was 29 weeks (27-30.1). In the group of women with SGA neonates, the z-scores of EFW were significantly lower than in the group with normal birthweight (0.1 ± 1.0 vs. -0.7 ± 0.9, *p* < 0.001) and the mean UtA PI was significantly higher (1.2 ± 0.4 vs. 1.4 ± 0.5, *p* < 0.001). Contrarily, no significant differencies in MAP were observed.

Moreover, MoM values of maternal serum PlGF were significantly lower (1.13 ± 0.9 vs. 0.70 ± 0.62, p < 0.001) and those of sFlt-1 as well as the sFlt-1/PlGF ratio significantly higher in the group of women who delivered SGA babies (0.9 ± 0.7 vs. 1.1 ± 0.8, p 0.037 and 1.8 ± 5.2 vs. 4.5 ± 6.8, *p* < 0.001, respectively) (Table 2).

### Multivariate regression model at third trimester

**Fig. 1:**
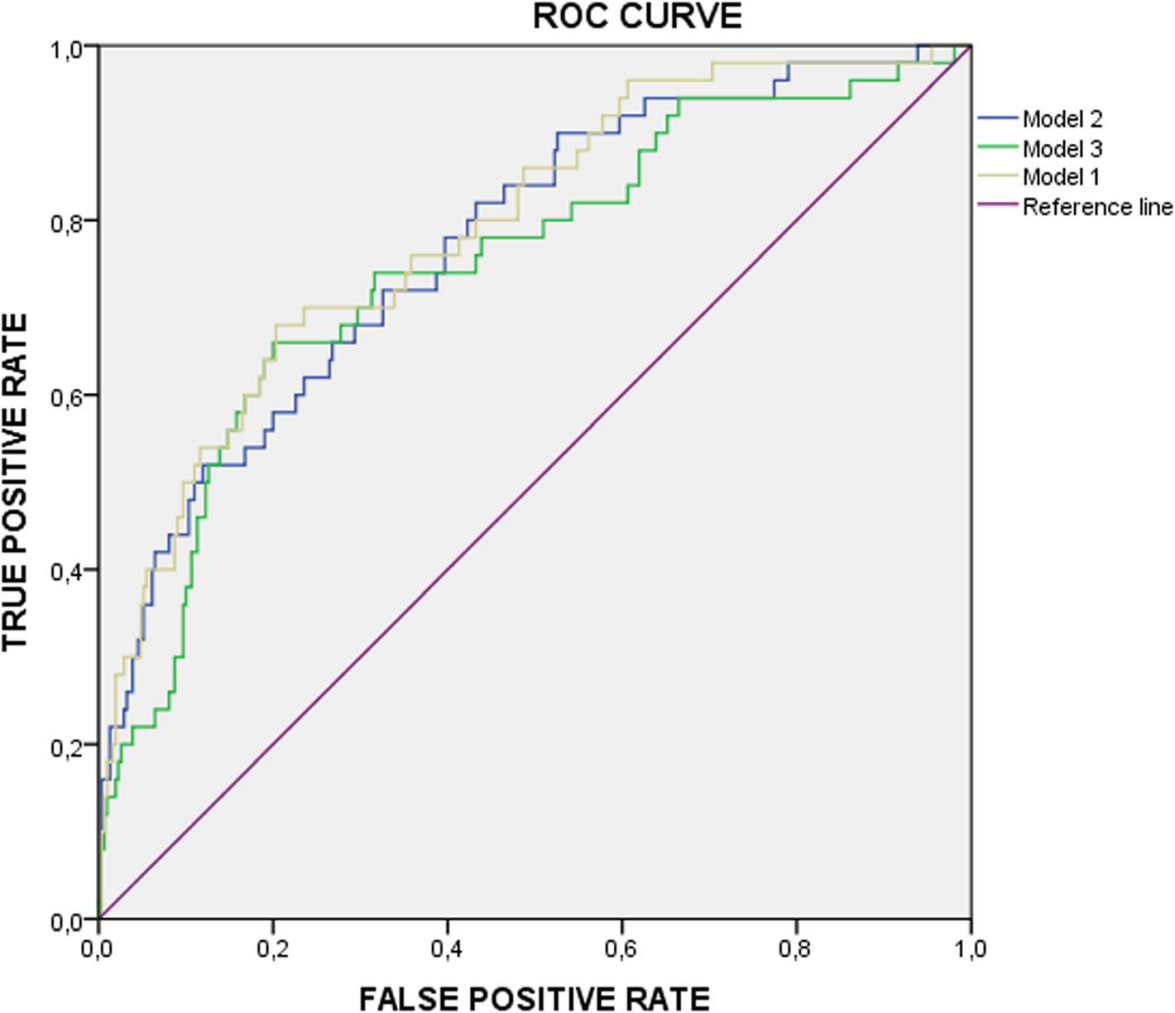
ROC for predictive models of SGA neonates at 27–30 weeks’ gestation in a high-risk population. Model 1: Maternal characteristics + EFW (z-scores) + mean UtA PI (MoM). Model 2: Maternal characteristics + PlGF (log MoM). Model 3: Maternal characteristics + EFW (z-scores) + mean UtA PI (MoM) + PlGF (log MoM). SGA, small-for-gestational age neonates; ROC curve, receiver-operating characteristics curve; EFW, estimated fetal weight; mean UtA PI, mean uterine artery Doppler pulsatility index; MoM, multiples of the median; PlGF, placental growth factor; log MoM, multiples of the median of the log10 for PlGF

A multivariate regression analysis demonstrated that the best prediction model at 27–30 weeks’ gestation was the combination of maternal characteristics, EFW (z-scores), mean UtA PI and PlGF with an AUC of 0.79 (95% CI, 0.72–0.86) (Fig. 1; Table 3). The detection rate (DR) for a 15% FPR was 56% with a negative predictive value (NPV) of 92.2%.

**Table 3:** Variables included in the third trimester predictive model

	OR (CI 95%)
Maternal age	0.98 (0.90–1.07)
Maternal weight	0.96 (0.92–0.99)
EFW (z-scores)	0.55 (0.31–0.82)
PlGF (log MoM)	0.59 (0.39–0.89)
Mean uterine artery PI (MoM)	3.04 (1.33–6.94)

*OR* Odds ratio, *CI* Confidence interval, *EFW* Estimated fetal weight, *PlGF* Placental growth factor, *log MoM* Multiples of the median of the log10 for PlGF, *MoM* Multiples of the median, *PI* Pulsatility index

The DR of a model including maternal characteristics, EFW (z-scores) and mean UtA PI (MoM) was 54% (AUC of 0.77 95% CI, 0.70–0.84 (FPR 15%)). When we evaluated the addition of PlGF at 27–30 weeks’ gestation, we found that a model that includes maternal characteristics and PlGF achieved similar detection of SGA neonates (DR of 56%, AUC of 0.75 95% CI, 0.67–272 0.83 (FPR 15%)) (Table 4).

**Table 4:** Screening performance for detection of SGA neonates at 27–30 weeks’ gestation

Model	AUC (95% CI)	DR at 15% FPR
1. Maternal characteristics + EFW (z-scores) + mean UtA PI (MoM)	0.77 (0.70–0.84)	54
2. Maternal characteristics + PlGF (log MoM)	0.75 (0.67–0.83)	56
3. Maternal characteristics + EFW (z-scores) + mean UtA PI (MoM) + PlGF (log MoM)	0.79 (0.72–0.86)	56

*SGA* Small-for-gestational age neonates, *AUC* Area under receiver-operating characteristic curve, *DR* Detection rate, *FPR* False-positive rate, *EFW* Estimated fetal weight, *UtA PI* Uterine artery Doppler pulsatility index, *PlGF* Placental growth factor, *log MoM* Multiples of the median of the log10 for PlGF, *MoM* Multiples of the median

## Discussion

### Main findings

This study shows that a model at early third trimester that combines maternal characteristics, EFW, uterine artery Doppler and PlGF achieves the detection of 56% of SGA neonates in a high-risk population of PE (FPR 15%), with a NPV of 92.2%. The performance of a model including maternal characteristics and PlGF is similar to a model including maternal characteristics and ultrasonic parameters (EFW and uterine artery Doppler).

### Comparison with previous studies

Some studies in the literature have evaluated the addition of biochemical markers to ultrasound at third trimester to increase the prediction SGA neonates achieving modest results. Bakalis et al. showed that a screening combining maternal characteristics and biophysical and biochemical markers at 30–34 weeks detects 57% of SGA fetuses delivering ≥ 37 weeks [11]. Miranda et al. showed a model that, combining maternal characteristics, EFW, maternal and fetal Doppler and biochemical markers, achieved a detection of 61% of SGA cases (FPR 10%) [12]. Triunfo et al. evaluated fetuses with EFW > 10th centile at 32–36 weeks. They showed that a combination of sFlt-1/PlGF ratio and EFW resulted in a DR of 66% of SGA neonates (for a FPR of 20%) [13]. These models show slightly better performance than our study, performed on a previously selected high-risk population according to 1st trimester PE screening.

In our study, the risk of delivering SGA neonates was increased in nulliparous women and those with lower BMI. With regard to first trimester parameters, MAP, PAPP-A and PlGF were lower and mean UtA PI increased in women delivering SGA babies. The relationship of lower MAP with SGA may be attributed to its positive correlation with BMI [25]. About mean UtA PI, maternal characteristics and biochemical markers, several studies have demonstrated differences on pregnancies with SGA neonates at 11–13 weeks [26–28]. However, it is well known that first trimester screening of SGA neonates performs poorer than the screening for Preeclampsia [29, 30].

When focusing on early third trimester, we found that pregnancies with SGA neonates had lower EFW and PlGF and higher values of mean UtA PI, sFlt-1 and sFlt-1/PlGF. This is in agreement with several reports that have provided evidence that angiogenic factors are different in late events [11, 12, 31–34]. However, while both biochemical markers showed significantly different concentrations between SGA and normally growing fetuses, PlGF was the only biochemical marker included in the model. This is consistent with previous studies that have demonstrated that decreased levels of PlGF are predictive of histological signs of underperfusion found in most pregnancies associated with FGR [35, 36]. Other studies haven’t found differences in sFlt-1 levels in pregnancies complicated with SGA without PE, suggesting that it is a more specific biomarker for maternal endothelial impairment associated to this latter condition [9, 37]. Consistent with these findings, Gaccioli et al. evaluated the effectiveness of the combination of fetal biometries and sFlt-1/PlGF ratio at 28 and 36 weeks in a low-risk population. They found that, at 28 weeks, PlGF and sFlt-1/PlGF were equally predictive for preterm SGA while sFlt-1 was a weaker predictor. This latter showed a sensitivity of 50% compared to 76.9% of PlGF and 73.1% of sFlt/PlGF respectively [38].

Cases of early growth restriction are specially associated with placental insufficiency and the associated changes in serum metabolites, which are linked to the severity of the disease. Herraiz et al. found that the sFlt1/PlGF ratio rised 4 weeks before the delivery of FGR fetuses, regardless of the presence of PE [39]. The low incidence of preterm SGA babies in our series (6 cases) could have blinded the utility of these parameters.

The evaluation of PlGF is an objective and feasible parameter that could be measured at bedside, avoiding problems related to operator depending ultrasound skills. Therefore, it could be a better option to detect high risk pregnancies in some settings where the possibility of performing a third trimester ultrasound is not available. Moreover, it could be part of a contingent screening: Evaluating PlGF as a first step and performing ultrasound only if it is altered. Some studies have proposed protocols that evaluate the risk of growth restriction at second trimester and propose a specific follow-up based on the results of this screening [40–42]. Triunfo et al. performed a third trimester scan in 50% of the population based on a second trimester screening achieving equivalent results to the strategy of performing a third trimester scan to the whole population (AUC 0.89 vs. 0.92) [40]. Poon et al. used a second trimester screening with biophysical (biometries, mean UtA PI) and biochemical (PlGF and alfa-fetoprotein (AFP)) markers to decide when to perform the third trimester scan. According to their results, 11% of the population required an ultrasound at 32 weeks and 44% at 36 weeks, with the objective to predict 80% of cases of SGA < 5th centile [41]. In a similar line, Lesmes et al. performed a second trimester screening combining fetal biometries and biochemical markers (PlGF, sFlt1, PAPP-A, free b-HCG and AFP), estimating that 11% and 46% of the population needed respectively to be reassessed at 32 and 36 weeks, to achieve an 80% detection of SGA < 5th centile [42].

We have no doubt that the way to detect the majority of pregnancies that will end with the birth of a SGA neonate, or at least to discard this possibility, goes through a combined model. The negative predictive value of our model (92.2%) is a remarkable aspect of the study. A model including PlGF may be useful as an additional tool for risk stratification classifying pregnancies as low or high risk of delivering late SGA neonates and, consequently, we may expect to rule out lower risk patients in order to avoid iatrogenic interventions and propose a specific plan for higher risk patients.

### Strengths and limitations

One strength of the study is that we evaluated a high-risk population of placental disease. We did not find studies that focus on such high-risk patients in which the prevalence of SGA neonates is expected to be higher. Another important issue is that the follow-up of the patients was strict and integrally made in our center and all our professionals had been trained to measure uterine Doppler PI and blood pressure.

On the other side, the limitations of our study are: first, that the population is very small with only 50 cases of SGA neonates. We believe that we would obtain better results in a larger population. Secondly, in our study the patients were evaluated by ultrasound at the beginning of the third trimester and this fact may decrease the effectivity of the model. It would be interesting to evaluate the model performing the third trimester assessment at 36 weeks. However, this strategy would have the disadvantage of limiting management alternatives. We do not consider prophylactic use of Aspirin a limitation of the study because it was not different between both groups. Indeed, it reflects the real clinical practice after the results of ASPRE trial [43]. According to this study, in high-risk women for PE by means first trimester screening, the use of aspirin reduces the incidence of preterm and early SGA by 20% and 40%, respectively. However, most cases of late SGA are not preventable.

## Conclusions

In conclusion, the performance of screening for SGA at early third trimester combining maternal characteristics, mean UtA PI, fetal biometry, MAP and biochemical markers in a high-risk population is poor. However, the high NPV may help to reduce maternal anxiety in this specific group of women at high risk of developing PE, change the policy of performing third trimester ultrasound in 100% of population and avoid iatrogenic interventions. Future studies are necessary to evaluate the performance of third trimester combined screening models, define specific protocols for high-risk pregnancies and evaluate if these protocols could reduce perinatal morbidity and mortality.

## Abbreviations

SGA: Small-for-gestational age neonates
FGR: Fetal growth restriction
PE: Preeclampsia
ART: Assisted reproduction technique
DM: Diabetes mellitus
CRL: Crown-rump length
PlGF: Placental growth factor
free β-hCG: Free β-human chorionic gonadotropin
PAPP-A: Pregnancy-associated plasma protein A
Ut A PI: Uterine artery pulsatility index
BP: Blood pressure
MAP: Mean arterial pressure
FPR: False positive rate
EFW: Estimated fetal weight
UA: Umbilical artery
MCA: Middle cerebral artery
CPR: Cerebroplacental ratio
DV: Ductus venosus
sFlt-1: Soluble FMS-Like Tyrosine Kinase-1
MoM: Multiples of the median
OR: Odds ratio
CI: Confidence interval
ROC: Receiver operating characteristics curve
AUC: Area under receiver–operating characteristics curve
BMI: Body mass index
DR: Detection rate
NPV: Negative predictive value
AFP: Alfa-fetoprotein
GA: Multiples of the median of the log10 for PlGF

## Availability of data and materials

The datasets used during the present study are available from the corresponding author on reasonable request.
